# The acclimatory response of the mayfly *Neocloeon triangulifer* to dilute conditions is linked to the plasticity of sodium transport

**DOI:** 10.1098/rspb.2022.0529

**Published:** 2022-07-27

**Authors:** Jamie K. Cochran, David B. Buchwalter

**Affiliations:** Department of Biological Sciences, North Carolina State University, Raleigh, NC 27695, USA

**Keywords:** mayfly, dilute, ion transport, acclimation, life history, sodium

## Abstract

Relative to a growing body of knowledge about the negative consequences of freshwater salinization, little is known about how aquatic insects respond to progressively ion-poor conditions. Here, we examined life-history and physiological acclimation in *Neocloeon triangulifer* by rearing nymphs from 1-day post-egg hatch to adulthood across a gradient of decreasing Na concentrations (15, 8, 4, 2 and 1 mg l^−1^ Na). We found no significant changes in survival, growth, development time and whole-body Na content across these treatments. Radiotracer data revealed that nymphs acclimated to their dilute exposures by increasing their rates of Na uptake and were able to maintain a relatively narrow range of uptake rates (±s.e.m.) of 38.5 ± 4.2 µg Na g^−1^ h^−1^ across all treatments. By contrast, the Na uptake rates observed in naive nymphs were much more concentration dependent. This acclimatory response is partially explained by differences in ionocyte counts on the gills of nymphs reared under different salinities. Acclimated nymphs were surprisingly less retentive of their sodium composition when subjected to deionized water challenge. By contrasting our findings with a previous *N. triangulifer* salinity acclimation study, we show a physiological affinity for dilute conditions in this emerging mayfly model.

## 1. Introduction

Freshwater ecosystems range widely in salinity, from practically deionized to ion-rich [1] as a function of geology, and can fluctuate with rainfall, evaporation and hyporheic influences [2,3]. It is currently unclear how natural freshwater salinity regimes shape aquatic communities and affect local biodiversity. Moreover, the relatively recent awareness that human activities (e.g. road deicing) negatively affect biodiversity via changes in the ionic composition of freshwaters requires that we deepen our understanding of how these changes affect the physiology and fitness of aquatic organisms [4–7]. While most attention is centered on increasing salinization, changes in precipitation, acid deposition and weathering are actually decreasing the major ion content of some freshwater systems [8,9]; [10, p. 296]; [11].

Aquatic insects are ecologically important and relied upon to make inferences about water quality and ecological conditions [12,13]; [14, p. 200]; [15]. Aquatic insects can be impacted by changes in freshwater salinity [6,16,17], which plays a role in determining where species can thrive [18,19]. While the response of aquatic insects to anthropogenically elevated ions in the field [6,16] has stimulated several laboratory-based efforts [17,20,21] to understand physiological mechanisms, relatively little is known about how insects cope with dilute conditions (but see [22]). Other studies have found lower abundances of taxa richness associated with low ionic concentration [23–25]. While some observations have suggested these patterns were caused by pH and nutrient availability [25,26], more recent studies in aquatic insects and fish suggest the cause may be the concentration of total dissolved solids [19,27–30].

Most aquatic insects are known to regulate a constant haemolymph osmolality despite the salinity of their external environment [31–33] via the constant turnover of ions. This maintenance affects the energy budgets of aquatic insects in waters with highly elevated major ions (leading to developmental delays and reduced growth) [34], which suggests reallocation of energy to maintain homeostasis [32,33,35,36]. As ion transport against concentration gradients is energy-intensive [37], similar reallocation could also be expected in extremely dilute water. Growth and reproduction in freshwater invertebrates [38,39] and fish [40] have been observed to increase at moderate salinities (compared to low salinities). This observation could be partly due to the high costs of osmoregulation in hyposaline waters. Most aquatic insects have mitochondria-rich osmoregulatory structures (such as ionocytes) on their body surface [31], which are used to take up ions from the surrounding water [35]. Ionocytes (also known as chloride cells) are sites of ion uptake on the body surfaces (especially tracheal gills) of nymphs [41]. Little is definitively known about how salinity impacts the density of ionocytes of aquatic insects [42–45].

To understand how changes in salinity affect the physiology and fitness of aquatic organisms, it is important to ask how well aquatic organisms can acclimate to changing salinity regimes. This question has been studied in daphnids [46,47] and mayflies [48,49], but only in terms of increasing salinity. Studies with the baetid mayfly, *Neocloeon triangulifer* (which has been recently established as a useful model for ecological [50], toxicological [51–56] and physiological [20,57,58] studies) have provided evidence of ion-specific physiological plasticity and ion-specific toxicity mechanisms in high-salinity waters [49]. However, we know little about the ability of *N. triangulifer* to acclimate to extremely dilute waters.

Here, we used *N. triangulifer* to ask how exposure to decreased major ions affects physiological responses and life-history outcomes across a gradient of dilute ionic conditions. We used a radiotracer approach to ask if Na transport rates of acclimated versus naive nymphs differed over a 15-fold range of Na concentrations. We also asked if changes to cuticular Na permeability resulted from prior exposure history by measuring the loss rates of ^22^Na from nymphs subjected to a deionized water challenge. Finally, ionocyte density was assessed for nymphs reared in dilute, standard and ion-rich waters to assess whether physiological differences (e.g. modified flux rates) may be due, in part, to observed morphological changes.

## 2. Methods

### (a) Mayfly husbandry and water preparation

*Neocloeon triangulifer* (WCC-2 clone) was originally isolated for culture from White Clay Creek (WCC), Chester County, PA, USA, (240 µS cm^−1^), and rearing methods were developed at the Stroud Water Research Center (SWRC; Avondale, PA) [50]. This clonal line of *N. triangulifer* has been maintained in artificial soft water (ASW) for several generations. Because *N. triangulifer* occupies habitats that are not typically sampled in biomonitoring programs, its salinity preferences are largely unknown. However, different clones of the species have been observed and collected ranging from 57 to 340 µS cm^−1^ (Dave Funk, 25 May 2022, personal communication), which is not as dilute as some of the treatments we used (table 1). *N. triangulifer* nymphs were reared in 1.8 l glass jars at room temperature (21–23°C) and a 14 : 10 h light : dark photoperiod. Seven replicate jars (with about 25 *N. triangulifer* nymphs each) for each condition were set-up and randomly spaced out on the bench top. Food was provided as periphyton grown on an acrylic plate (gifted by collaborators at SWRC). Periphyton plates were grown by allowing fresh stream water from WCC, PA to flow over the plates continuously for 2–4 weeks (as described previously by [59] and [60]). Then, one periphyton plate was immediately added to each treatment water, and a second supplementary plate was added two weeks into rearing. All jars were gently aerated for the entire experiment to maintain oxygen saturation.

**Table 1.** Water chemistry for all experimental waters. Conductivity is reported in µS cm^−1^. Ions and TDS are all reported in mg l^−1^. All waters were sampled, filtered and verified by NC State University's Environmental and Agriculture Testing Services Lab (ICP-EATS). Measured values (when available) are reported in parentheses beside nominal values. Concentrations were within 15% of nominal values, except for measurements marked by an asterisk, which had between 18 and 33% error.

treatment	conductivity	pH	TDS	Na	total S (as SO_4_)	Ca	Mg	K	Cl	CO_3_
high salinity	777	7.6	552.5	157 (152)	7.8	12.7	3.4	1.4	255	42.6
control (ASW)	131	7.4	131.3	15.0 (15.5)	7.8 (7.3)	12.7 (10.8)	3.4 (3.1)	1.4	14.1	42.6
½ control	67.8	7.4	59.8	7.5 (7.9)	3.9 (4.5)	6.4 (4.5)*	1.7 (1.9)	0.7	7.1	21.3
¼ control	34.4	7.2	33.2	3.8 (4.0)	1.9 (2.6)*	3.2 (3.6)	0.9 (1.1)*	0.4	3.5	10.7
1/8 control	18.9	7.2	18.9	1.9 (1.9)	0.9 (0.8)	1.6 (1.6)	0.4 (0.6)*	0.2	1.8	5.3
1/16 control	9.8	7.1	10.4	0.9 (0.9)	0.5 (0.4)	0.8 (0.9)	0.2 (0.3)*	0.1	0.9	2.7
DI	1.1	7.0	0.5	−(<0.1)	−(<0.1)	−(<0.02)	−(<0.01)	—	—	—

ASW is our routine culture media and was the control water, as well as the base water diluted for these experiments. To create more dilute waters, ASW was diluted using reverse-osmosis deionized water to create the desired concentration (table 1). The ‘high-salinity’ water was prepared as in [49] and only used in rearing for ionocyte staining.

### (b) Life-history outcomes

We designated four of the seven replicate jars to be used for collecting life-history data (survival, final subimago weight and development time). Exposures lasted 24–33 days, with subimagos emerging over a 9-day period for all conditions. As they emerged in the late afternoon, subimagos were collected into a mesh-lined collection lid. Subimagos were immediately collected, placed in clean, labelled 1.5 ml microcentrifuge tubes, and stored frozen (−20°C) before wet weights were obtained. Life-history outcomes were compared among groups as the mean value of each response variable from each jar using a one-way ANOVA with Tukey's multiple comparisons test using GraphPad Prism (v6, GraphPad Software, La Jolla, CA, USA). All data were also analysed for normality.

### (c) Whole-body sodium content

We collected nymphs (around 22–24 days old) to be analysed for their whole-body sodium content. Nymphs were dried overnight at 60°C and subsequently weighed. Dried nymphs were microwave digested (CEM MARSXpress) in 1 ml Omnitrace Ultra High Purity Nitric Acid (EMD Chemicals, Darmstadt, Germany). NC State University's Environmental and Agriculture Testing Services Lab analysed samples via ICP-OES (Department of Soil Science, North Carolina State University, Raleigh, NC, USA) to determine the whole-body concentration of sodium. Quality control blanks were below Na detection limits. Measured sodium concentrations in certified reference material (freeze-dried NIST 2976-mussel tissue) were within 10% of the expected concentrations. Whole-body sodium measurements were compared among groups using a one-way ANOVA with Tukey's multiple comparisons test using GraphPad Prism. All data were also analysed for normality.

### (d) Ion flux experiments

We used nymphs from three of the seven replicate jars for ion flux experiments. Nymphs were 21 days old for all ^22^Na uptake experiments and 23 days old for all ^22^Na loss experiments. Radioactive experimental waters were made with ASW, and appropriate dilutions spiked with ^22^NaCl (PerkinElmer, Billerica, MA, USA). Exposure activities ranged from 135 to 220 Bq ml^−1^. Exposures were measured with PerkinElmer Wallac Wizard 1480 Automatic Gamma Counter (Shelton, CT) immediately before the experiments began.

Uptake and loss rates were calculated using the slopes of linear regression analysis using GraphPad Prism. Mass-specific calculations were based on wet weights. Only measurements with counting errors less than 10% were used in analyses. Flux rates were compared among groups using a one-way ANOVA with Tukey's multiple comparisons test using GraphPad Prism. Data were also analysed for normality.

#### (i) Uptake rate

Individual nymphs were placed into 24 100 ml high-density polyethylene beakers with 15 ml of radioactive exposure water. All experiments had eight spatially randomized replicates per 3, 6, and 9 h time point. All beakers were gently aerated and sealed with ParaFilm for the entire experiment. At each time point, nymphs were removed from the radioactive exposure waters by gently pipetting them into a mesh strainer (collecting any residual radioactive water in a waste container) and gently blotting dry. The nymphs were then rinsed in two consecutive water baths of the corresponding unlabeled exposure water to remove loosely adsorbed ions from the exoskeleton. Each individual nymph was then placed in a 20 ml glass vial with 3 ml of the corresponding unlabelled exposure water, and counted with the PerkinElmer Wallac Wizard 1480 Automatic Gamma Counter (Shelton, CT) for 3 minutes. After counting the 3- and 6 h time points, nymphs were returned to their original experimental cup, so that we could serially measure uptake in the same nymph across the full experiment.

#### (ii) Sodium loss rates

To assess whether the acclimation response included changes in the retentiveness of ions (either through enhanced resorption of ions from urine in the hindgut or reduced permeability of the cuticle), we measured the loss of ^22^Na from nymphs exposed to deionized water challenge. Nymphs (21 days old) were labeled with ^22^Na by placing them in a 200 ml high-density polyethylene beaker with 25 ml radioactive experimental water (corresponding with their unlabeled rearing water) for 48 h to acquire a strong ^22^Na signal (average 14 Bq per mg insect wet weight). All beakers were gently aerated for the entire experiment and sealed with ParaFilm. At 48 h, nymphs were removed from the radioactive exposure waters by gently pipetting into a mesh strainer (collecting any residual radioactive water in a waste container) and blotting dry. The nymphs were then rinsed in two consecutive water baths of the corresponding exposure water to remove loosely adsorbed ions from the exoskeleton, before being placed in a 20 ml glass vial with 3 ml of deionized water and counted with the PerkinElmer Wallac Wizard 1480 Automatic Gamma Counter (Shelton, CT) for 3 min. Each nymph was then placed in a 100 ml high-density polyethylene beaker with 100 ml of deionized water. At 3 and 9 h, nymphs were removed from the experimental cup by gently pipetting into a 20 ml glass vial with 3 ml of deionized water and counted with the PerkinElmer Wallac Wizard 1480 Automatic Gamma Counter (Shelton, CT) for 3 min. The experimental cups were refreshed with 100 ml of fresh deionized water, while the nymphs were counted to minimize re-uptake of lost ions. The individual nymphs were followed across the full experiment.

### (e) Ionocyte staining

A 2% AgNO3 solution was applied to a single live *N. triangulifer* nymph. After resting in direct light for 5 min, gills originating from abdominal segments four or five were plucked off the live nymph and imaged immediately on a Leica MZ 16 F stereoscope. The size of each gill was measured using Lecia Application Suite X (LAS X) for Life Science. The number of ionocytes was manually counted for each gill by looking for dark spots approximately 32 µm in diameter and making a mark through each counted ionocyte to ensure none were counted more than once.

## 3. Results

### (a) Life history

Life-history outcomes (table 2) were not significantly impacted by chronic exposure to dilute water. Across all exposure conditions, mean days to emergence was 26.4 ± 0.2, mean survival was 88.6 ± 2.2%, and mean subimago wet weight was 4.0 ± 0.1 mg (mean ± s.e.m.).

**Table 2.** Summary of life-history outcomes (including mean days to emergence, mean per cent survival, mean subimago weights (mg) and mean whole-body sodium (±s.e.m.).

exposure condition (mg l ^−1^ Na)	mean days to emergence	mean survival (%)	mean subimago mass (mg)	mean whole-body sodium (μg Na mg dry wt^−1^)
15	27 ± 0.2	86 ± 3.3	4.1 ± 0.1 (*n* = 84)	6.0 ± 0.4 (*n* = 16)
8	27 ± 0.2	83 ± 3.8	4.0 ± 0.1 (*n* = 68)	6.2 ± 0.2 (*n* = 9)
4	26 ± 0.2	87 ± 2.8	4.4 ± 0.1 (*n* = 72)	5.5 ± 0.3 (*n* = 10)
2	26 ± 0.2	91 ± 3.0	3.6 ± 0.2 (*n* = 68)	5.5 ± 0.8 (*n* = 8)
1	26 ± 0.2	96 ± 1.6	3.8 ± 0.1 (*n* = 70)	6.2 ± 0.4 (*n* = 8)

### (b) Whole-body sodium content

Sodium concentrations were not different between rearing conditions. Sodium concentrations were 5.9 ± 0.16 (mean ± s.e.m.) across all rearing conditions (table 2).

### (c) Ion flux

#### (i) Uptake rates

**Figure 1.**
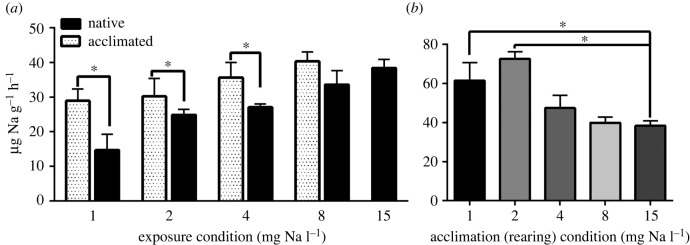
Sodium uptake rates were calculated from 9 h time course experiments. (*a*) Sodium uptake rates in naive *N. triangulifer* nymphs (black bars) and ‘acclimated’ nymphs reared under different water conditions (dotted bars). (*b*). Sodium uptake rates of ‘acclimated’ nymphs transferred to standard condition water (15 mg l^−1^) (mean ± s.e.m.). Asterisks represent significant differences between groups (*p* = 0.0001–0.0140).

Sodium uptake rates were concentration dependent for naive nymphs (reared in control water prior to acute exposures to dilute waters) (figure 1*a*). For example, naive nymphs had a sodium uptake rate of 38.4 ± 2.5 µg Na g^−1^ h^−1^ in control water, but a smaller sodium uptake of 14.6 ± 4.6 µg Na g^−1^ h^−1^ in 0.9 mg l^−1^ sodium water (figure 1*a*). Sodium uptake rates were markedly increased in nymphs chronically reared under more dilute conditions relative to naive reared nymphs. For example, acclimated nymphs had an approximately 50% higher uptake rate than naive nymphs in the lowest sodium water (0.9 mg l^−1^) (*p* = 0.0004) (figure 1*a*). These elevated Na transport rates in acclimated nymphs were within 24% of the uptake rates in control nymphs. When acclimated nymphs were transferred to control water, we observed a marked increase in Na transport. For example, nymphs reared in 0.9 mg l^−1^ water had a 2.4-fold higher sodium uptake rate in 15 mg l^−1^ water than in 0.9 mg l^−1^ water (figure 1*b*).

#### (ii) Sodium loss rates

**Figure 2.**
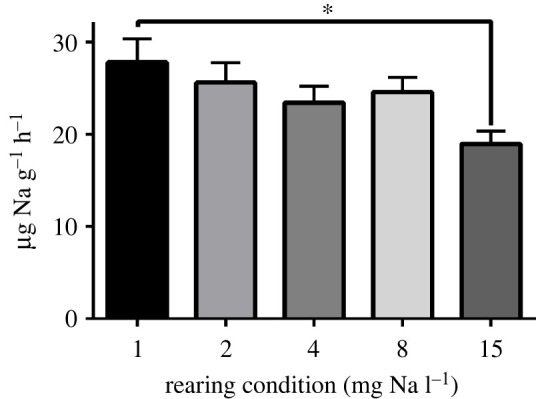
Sodium loss rates of *N. triangulifer* nymphs reared under different water conditions over 9 h exposure to deionized water (mean ± s.e.m.). Asterisks represent significant differences between groups (*p* = 0.0188).

We found that the rate of Na loss from nymphs subjected to deionized water challenge was differed by exposure history. For example, naive nymphs reared in 15 mg Na l^−1^ water had a loss rate of 19.0 ± 1.4 µg Na g^−1^ h^−1^ in deionized water, whereas nymphs chronically reared in 0.9 mg l^−1^ had a loss rate of 27.9 ± 2.5 µg Na g^−1^ h^−1^ (*p* = 0.0188) (figure 2).

### (d) Ionocyte staining

**Figure 3.**
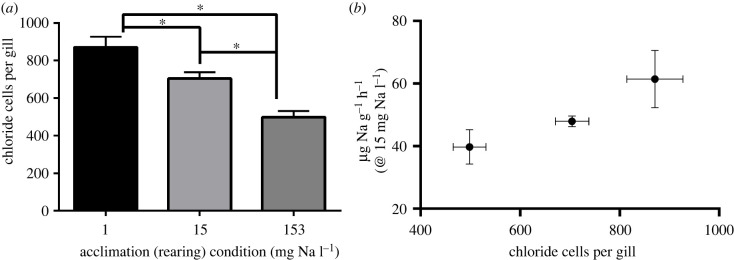
(*a*) Number of ionocytes on gills of nymphs reared in dilute (1 mg Na l^−1^) control (15 mg Na l^−1^) and ion-rich (153 mg Na l^−1^) water. (*b*) Relationship between the number of ionocytes per gill and the measured sodium uptake rate in standard condition water (15 mg Na l^−1^) in chronically reared nymphs. Uptake rate for high-salinity treatment (square) comes from [49]. *R*^2^ = 0.96 (mean ± s.e.m.). Asterisks represent significant differences between groups (*p* = 0.0001–0.0188).

The number of ionocytes on *N. triangulifer* gills varied with Na concentration (figure 3*a*) [49]. Nymphs reared in 15 mg Na l^−1^ had 676 ± 40.5 ionocytes per gill, nymphs reared in 1 mg Na l^−1^ had 794 ± 37.9 ionocytes per gill, and nymphs reared in 153 mg Na l^−1^ had 382 ± 32.9 ionocytes per gill (mean ± s.e.m.). A positive relationship was observed between the number of ionocytes per gill and the Na uptake rates of nymphs exposed to standard condition water (15 mg Na l^−1^) (*R*^2^ = 0.96) (figure 3*b*). A two-tailed unpaired *t*-test showed no significant difference between the number of ionocytes on gills from abdominal segments four or five, regardless of treatment. The sizes of the gills did not differ significantly based on treatment. See electronic supplementary material, figure S1 for the full complement of gill images.

## 4. Discussion

Remarkably little is known about the osmoregulatory physiology of aquatic insects. Since aquatic insects are routinely used as bio-indicators in natural systems [12–15], it is imperative that we develop a more robust understanding of the physiological determinants of realized salinity niches. In streams with lower ionic concentrations, some taxa are more prevalent than others [29]. Some suggest these patterns in distribution were caused by pH and nutrient availability [25,26], but later studies suggest the cause may be the concentration of total dissolved solids [19,29,30], though none exclude either hypothesis. This natural complexity has led us to do controlled physiological experiments to study how insects respond to salinity [21,34,35,61]. Only recently have we begun to explore the importance of acclimation and physiological plasticity in these responses [49]. Here we show, for the first time, a basis for physiological affinity (via acclimation) to dilute conditions.

Our finding suggests that *N. triangulifer* nymphs are strong regulators and tightly control whole-body sodium content, even in ion-poor rearing waters (table 2). This finding is commensurate with other studies in *N. triangulifer*, where total body sulfur [34] and sodium [61] content were strongly regulated across a gradient of increased sulfate and sodium concentrations, respectively. Further, Patrick *et al*. [62] showed that the mosquitoes *Aedes aegypti* (Linnaeus 1762) and *Culex quinquefasciatus* (Say 1823) both maintained high haemolymph NaCl concentrations despite being reared in dilute media for multiple generations. However, it is important to note that all ions in ASW were diluted, so any effects can thus not necessarily be assigned exclusively to Na concentration.

Additionally, *N. triangulifer* nymphs exhibited no difference in their growth rates, development time and survival across a 15-fold gradient of sodium concentrations (table 2). This finding differs from other studies in freshwater invertebrates [38,39,63] and fish [40], where growth and survival rates were negatively influenced by lower salinities. We note, however, that these other species are not known to be typically abundant in ion-poor water (IPW), whereas many EPT species typically thrive in such conditions. This suggests that *N. triangulifer* is physiologically adaptable to dilute conditions.

We provide two lines of evidence for physiological acclimation in *N. triangulifer* which allowed the nymphs to maintain fitness over a wide range of salinities. First, when nymphs were subjected to as much as a 15-fold decrease in ambient sodium concentrations, acclimated animals had uptake rates that were only 24% lower than controls. By contrast, naive nymphs were 62% different from controls (figure 1*a*). Second, when we performed the reciprocal cross of dilute-acclimated animals to control conditions, we observed a 40% increase in sodium uptake rates (figure 1*b*). Both results demonstrate that the acclimation process includes a significant ability to upregulate sodium transport. Nguyen & Donini [22] showed that acute exposure to IPW in a larval midge, *Chironomus riparius*, increased the uptake of Na^+^, Cl^−^ and H^+^ by the anal papillae, whereas long-term exposure to IPW resulted in increased anal papillae size but no increase in ion uptake. Another study in *Aedes aegypti* found that transport by the anal papillae in mosquito larvae is based on external salinity (Na^+^ and Cl^−^ uptake decrease with higher salinities, relative to lower salinities) [64]. Durant *et al*. [65] showed that Daphnia survival in low Ca lakes may be managed by limiting Ca loss and increasing Ca^2+^ uptake to maintain free ionic Ca^2+^ in the haemolymph. Harris & Santos [66] showed that the mangrove crab *Ucides cordatus* increased Na uptake relative to controls after acclimation to a hypo-osmotic medium. Other studies have found that increased salinity impacts the uptake rates of sulfate in freshwater mussels [67], and calcium in rainbow trout, [68] and tilapia [69].

These findings complement a previous study [49], where *N. triangulifer* reared in water with elevated major ion concentrations were able to decrease sodium and sulfate uptake rates relative to naive nymphs. That study demonstrated that nymphs reared under ion-rich conditions attempted to evade excessive ion uptake, as they had markedly lower uptake rates than naive nymphs. Since the present study uses animals from the same biological population as Orr *et al*., we can for the first time provide insights into the relative acclimation potential of a mayfly to both dilute and ion-rich conditions. Orr *et al*. report that nymphs acclimated to a 10.2-fold increase in ambient Na reduced their Na uptake by about 50% relative to naive animals [49]. In our present study, nymphs acclimated to a 15-fold decrease in Na increased their Na uptake by about 49% relative to naive animals (figure 1*a*). While the magnitude of the acclimatory response is similar in this comparison, the flux rates resulting from those changes are quite different. On the dilute end of the spectrum, the sodium uptake rate of acclimated animals was only 24% lower than control animals (figure 1*a*) whereas on the ion-rich end of the spectrum, acclimated animals had flux rates that were 47% higher than controls. Thus, acclimated nymphs maintain influx rates closer to those associated with control conditions under dilute scenarios than they do after acclimation to ion-rich conditions.

One possibility is that the dilute-acclimated nymphs maintain a sufficiently positive net Na influx (see electronic supplementary material, figure S2) to meet the physiological demands associated with the life-history outcomes observed here (table 2). Another possibility is that if the differences in apical influx rates between control nymphs and dilute-acclimated nymphs are somehow detrimental physiologically, then it must be offset by either (i) reduced turnover (efflux) or (ii) by enhanced dietary acquisition of Na from periphyton. For this latter possibility to be true, the acclimated nymphs would have to enhance their dietary assimilation of Na from periphyton, and the periphyton itself would have to maintain somewhat consistent Na concentrations under very dilute scenarios. Regardless of which of the above possibilities is correct, it is clear that the enhanced apical Na uptake observed here in dilute-acclimated nymphs plays a key role in their ability to better tolerate ion-poor than ion-rich conditions. Together, these observations provide a physiological explanation for why some species perform better under dilute conditions, relative to salinized conditions.

We hypothesized that part of the acclimatory response to extremely dilute conditions could be enhanced ion retention. This hypothetically could occur via cuticular changes to reduce the diffusive loss of ions, or via enhanced resorption (rescue) of ions from the dilute urine (see [41]). Because physiological loss of sodium (or other ions) is commensurate with uptake rates in tightly regulating organisms [61], we subjected the nymphs to deionized water to obtain an unbiased comparison of Na retention. Since our measurements of whole-body sodium did not differ across treatments, the concentration gradients between the nymphs and deionized water were the same across treatment groups. We were surprised that nymphs reared under ion-poor conditions were less retentive (lost sodium faster) in deionized water than nymphs reared in control water (figure 2). Thus, our hypothesis was not supported by our data. Other studies in ion-poor conditions have found that decreasing paracellular permeability is key in reducing passive loss of ions in both goldfish [70] and euryhaline teleosts [71]. It is possible that differences in the sodium loss rates we observed are related to ionocyte numbers.

Though we only counted ionocytes in nymphs reared under three different conditions, we observed a marked negative association between salinity and ionocyte numbers (figure 3*a*). Our results agree with previous observations from Wichard *et al*. [45] where prolonged exposure to dilute freshwater in the laboratory resulted in more ionocytes than those reared in concentrated or normal freshwater in the mayfly *Callibaetis coloradensis.* These cells were more widely distributed along the whole gill than those in the other conditions. The same study found a similar relationship in *Callibaetis floridanus* nymphs field collected from fresh and brackish water [45]. Exposure to ion-rich water resulted in degeneration of ionocytes in the stonefly *Paragnetina media*, suggesting a morphological response to the ion-rich water [72]. Kefford *et al*. [73] showed that the surface area of the anal papillae was impacted by salinity treatments in *Chironomus oppositus* larvae; however, it was found that factors other than salinity impacted the size of the anal papillae in other larval chironomids. Other studies have not observed differences in ionocyte numbers as a function of environmental conditions. For example, Nowghani *et al*. found no impact of similar but sub-lethal levels of NaCl on ionocyte density in the mayfly *Hexagenia rigida* [74]; however, this study was conducted on a relatively shorter timescale (7 days). Berrill *et al*. [75] did not observe differences in ionocyte densities in a variety of mayfly species associated with pH stress. It is important to note the limitations associated with counting the ionocytes by hand, as it is not possible to distinguish between cells that are on the surface and exposed to the external environment, and those that are not [31]. Further, this technique does not allow us to assess whether the surface area of each ionocyte may have changed due to other conditions. Overall, we think that while the count of ionocytes is imperfect, it nonetheless provides insight into a morphological change associated with physiological acclimation.

Together, our data suggest that *N. triangulifer* nymphs have a strong ability to acclimate to dilute conditions. This acclimation is largely driven by plasticity in ion uptake rates and is supported by a proliferation of ionocytes. We suspect that the ability to thrive in extremely dilute conditions requires the maintenance of uptake rates that exceed the loss via cuticular leakiness and insufficient rescue of ions from dilute urine [41,76] (see electronic supplementary material, figure S1). Through acclimation, *N. triangulifer* can maintain a relatively narrow range of ion transport rates even under extremely dilute conditions. By comparing our results to those of Orr *et al*. [49], we also see that this organism is physiologically more likely to thrive in dilute environments than in ion-rich environments. How broadly this applies to other taxa is yet to be explored.

## Data Availability

Data available from the Dryad Digital Repository: http://dx.doi.org/10.5061/dryad.2280gb5v3 [77]. Electronic supplemental material is available online [78].
